# Air-powder polishing on self-ligating brackets after clinical use: effects on debris levels

**DOI:** 10.1590/2177-6709.21.5.090-094.oar

**Published:** 2016

**Authors:** Mônica L. S. Castro Aragón, Leandro Santiago Lima, David Normando

**Affiliations:** 1 Graduate Program in Dentistry, Universidade Federal do Pará (UFPA), Belém, Pará, Brazil.; 2 Private practice, Belém, Pará, Brazil.; 3 Adjunct professor, Universidade Federal do Pará (UFPA), School of Dentistry, Belém, Pará, Brazil. Coordinator, Universidade Federal do Pará (UFPA), Postgraduate Program in Dentistry, Belém, PA, Brazil.

**Keywords:** Orthodontic brackets, Ceramics, Sodium bicarbonate, Dental prophylaxis

## Abstract

**Introduction:**

Debris buildup on brackets and arch surfaces is one of the main factors that can influence the intensity of friction between bracket and orthodontic wire.

**Objective:**

This study sought to evaluate the effect of air-powder polishing cleaning on debris levels of self-ligating ceramic brackets at the end of orthodontic treatment, compared to the behavior of conventional brackets.

**Methods:**

Debris levels were evaluated in metal conventional orthodontic brackets (n = 42) and ceramic self-ligating brackets (n = 42) on canines and premolars, arranged in pairs. There were brackets with and without air-powder polishing. At the end of orthodontic treatment, a hemiarch served as control and the contralateral hemiarch underwent prophylaxis with air-powder polishing. Debris buildup in bracket slots was assessed through images, and Wilcoxon test was used to analyze the results.

**Results:**

The median debris levels were statistically lower in the conventional metal brackets compared to self-ligating ones (*p* = 0.02), regarding brackets not submitted to air-powder polishing. Polishing significantly reduced debris buildup to zero in both systems, without differences between groups.

**Conclusions:**

Ceramic self-ligating brackets have a higher debris buildup in comparison to conventional metal brackets *in vivo*, but prophylaxis with sodium bicarbonate jet was effective in reducing debris levels in self-ligating and also in conventional brackets.

## RESUMO

**Introdução:**

o acúmulo de detritos nas superfícies de braquetes e arcos é um dos principais fatores que podem influenciar na intensidade do atrito entre o braquete e o fio ortodôntico.

**Objetivo:**

o presente estudo procurou avaliar o efeito da limpeza com jatos de bicarbonato de sódio nos níveis de detritos em braquetes autoligáveis cerâmicos ao final do tratamento ortodôntico, em comparação com o comportamento dos braquetes convencionais.

**Métodos:**

foram avaliados os níveis de detritos em braquetes convencionais de metal (n = 42) e braquetes autoligáveis cerâmicos (n = 42) de caninos e pré-molares, dispostos em pares. No final do tratamento ortodôntico, uma hemiarcada serviu como controle e a hemiarcada contralateral foi submetida à profilaxia com jatos de bicarbonato de sódio. O acúmulo de detritos nas canaletas dos braquetes foi avaliado por meio de imagens, e o teste de Wilcoxon foi utilizado para analisar os resultados.

**Resultados:**

a mediana do nível de detritos foi estatisticamente menor nos braquetes convencionais, quando comparados aos autoligáveis (*p* = 0,02). O jateamento reduziu o nível de detritos a zero, em ambos os sistemas, sem diferenças entre os grupos.

**Conclusões:**

os braquetes autoligáveis cerâmicos apresentaram, *in vivo*, maior acúmulo de detritos do que os braquetes convencionais; porém, a profilaxia com jatos de bicarbonato de sódio foi efetiva na redução dos níveis de detritos tanto nos braquetes autoligáveis quanto nos convencionais.

## INTRODUCTION

Self-ligating brackets have emerged in order to speed the progress of orthodontic treatment up by means of reducing friction levels^1^ and eliminating the need for ligatures used in conventional brackets. However, debris buildup on brackets and arch surfaces is one of the main factors that can influence the intensity of friction between bracket and orthodontic wire.^2^ ^,^ ^3^ Although it is stated that one of the advantages of self-ligating brackets is decreased dental plaque buildup,^4^ there is no substantial evidence to support this idea.

Bonding a fixed orthodontic appliance increases biofilm retention and hinders teeth cleaning practices.^5^ ^,^ ^6^ ^,^ ^7^ As a complement to patient's oral health, some prophylactic techniques are used by professionals during treatment maintainence,^8^ with air-powder polishing being noteworthy.^9^ Its effectiveness in removing dental plaques and stains has been widely reported^10^ ^,^ ^11^ and it has been increasingly used. This technique requires less physical effort, short clinical period for execution, and does not generate heat compared with rubber cup or Robson brush and prophylactic paste,^8^ ^,^ ^10^ in addition to producing less risk of damage to the fixed orthodontic appliance.^8^

The investigation of changes in debris buildup during orthodontic treatment with fixed appliances could lead to better means to prevent or at least reduce the risks associated with such treatment, such as increased friction,^12^ ^,^ ^13^ enamel demineralization^4^ and bacterial adhesion.^14^ Despite some studies examining the effects of cleaning orthodontic wires^12^ and conventional metal brackets, ^13^ efficient methods employed to clean orthodontic self-ligating brackets have not yet been compared to conventional ones: Are there differences between cleaning metal or ceramics brackets? Does the cleaning efficiency of self-ligating brackets differ from that of conventional ones? Given the importance of keeping the bracket-wire system clean, this study sought to evaluate the effect of air-powder polishing cleaning on debris levels of self-ligating ceramic brackets at the end of orthodontic treatment, compared to conventional brackets.

## MATERIAL AND METHODS

The present study was approved by the Ethics Committee of Universidade Federal do Pará (UFPA) with registration n° 157.182. All participants, or their legal guardians in cases of minors, signed an informed consent form (ICF) at baseline. 

Sample size calculation was performed assuming that air-powder polishing on orthodontic brackets after clinical use would modify the levels of debris and friction at 0.2 N, with a power of 80%, a two-tailed alpha of 5%, and the standard deviation of difference of 0.3 N. The standard deviation of difference was determined by a pilot study with nine pairs of conventional brackets at the end of orthodontic treatment. Sample sizes were determined to be 20 in each group.

The effects of air-powder polishing on preadjusted brackets were evaluated immediately after removal in 84 brackets: 42 conventional ones (0.022 x 0.028-in slot, straight-wire, Mini Diamond^TM^, Ormco, Glendora, California, USA) and 42 ceramic self-ligating ones (slot 0.022 x 0.028-in, straight-wire, QuicKlear^TM^, Forestadent, Pforzheim, Baden Württemberg, Germany). Each bracket system was divided into 21 pairs, comprising a bracket without blasting (control group) and another blasted. Groups were selected by simple randomization. Mean treatment duration was of 26.19 months. The following sequence of wires was used: 0.014-in, 0.016-in, 0.018-in (NiTi), 0.020-in steel, finishing with 0.019 x 0.025-in steel wire.

The sample comprised brackets from patients of a private orthodontic practice who were treated by the same orthodontist performing routine prophylaxis for patients at every visit. At the end of orthodontic treatment, and at the time of bracket removal, the finalization archwire was carefully removed and the brackets of the maxillary hemiarch and lower arch were cleaned by air-powder polishing (Ayron, Maquira, São Paulo, Brazil). A handpiece (Practical Jet Kondortech, San Carlos, Brazil) was used for blasting during 5 seconds at a 3-mm distance, forming an angle of 90° to the surface of each bracket.^9^ The contralateral quadrants did not undergo prophylaxis and served as control. 

The corresponding brackets of canines and premolars of the four quadrants were removed. Ligature cutting pliers were used, with gentle pressure applied across the interface between the bracket base and the adhesive. This technique was chosen, so as to not produce significant deformations to the brackets.^15^ Nevertheless, when removed, the structural integrity of brackets was evaluated, and those with defects or fractures were eliminated from the study. Lastly, each retrieved bracket was fixed onto premade acrylic plates. 

In order to perform the debris index, images of each bracket slot were obtained with the aid of a digital microscope (MV1302U-PL Miview USB Microscope, Cosview, Shenzhen, Guangdong, China), under magnification of 120x. Each image was checked by a single examiner with experience in the method, and received scores according to the presence of debris on the bottom surface of the bracket slot.^16^ For analysis of error, a second reading for all images was made by the same operator. Spearman correlation was used to check for reproducibility. The following scores were assigned: 0 - total absence of debris; 1 = low presence of debris in less than 25% of the surface area of the slot; 2 = moderate presence of debris, occupying more than 25% and less than 75% of the surface area of the slot; and 3 = enhanced presence of debris, occupying more than 75% of the surface area of the slot. 

Median and interquartile range were obtained for debris. Wilcoxon test was used to compare the levels of debris between groups. Data were analyzed with Bioestat v.5.3 software (Institute of Sustainable Development Mamirauá, Belém, Pará, Brazil). The confidence level employed was 95%.

## RESULTS

Spearman correlation showed excellent reproducibility (*p* < 0.0001) for the debris scores obtained in this study (*r* = 0.96). After bracket removal, the median debris level was significantly lower for conventional brackets (med = 1.0) when compared to self-ligating brackets (med = 2.0; *p* = 0.02).

Cleaning with air-powder polishing blasting was effective in removing debris in both conventional brackets (*p* < 0.0001) and self-ligating ones (*p* = 0.0001), taking the median to zero. After blasting, there was no difference in the levels of debris between the types of brackets (*p* = 0.43; Table 1).

**Figure 1:**
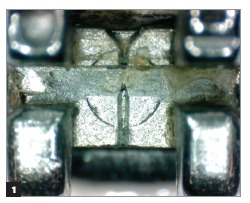
Conventional bracket after clinical use not subjected to prophylaxis with air-powder polishing.

**Figure 2:**
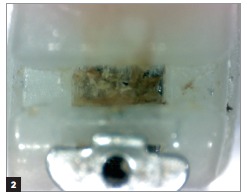
Self-ligating bracket after clinical use not subjected to prophylaxis with air-powder polishing.

**Figure 3:**
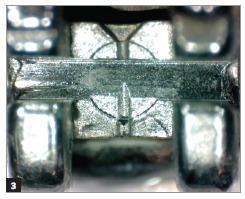
Conventional bracket after clinical use subjected to prophylaxis with air-powder polishing.

**Figure 4:**
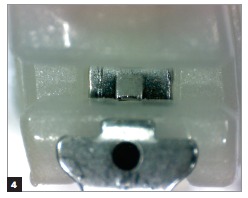
Self-ligating bracket after clinical use subjected to prophylaxis with air-powder polishing.

**Table 1:** Median, interquartile range (IQR) and *p* value for level of debris of both groups, before (T_1_) and after air-powder polishing cleaning (T_2_).

	Conventional		Self-ligating		*p*-value
	Median	IQD	Median	IQD	
T_1_	1	1	2	1	0.02
T_2_	0	0	0	1	0.43*
p-value (bilateral)	< 0.0001		0.0001		---

* non-significant.

## DISCUSSION

Biofilm and debris buildup in the slot of orthodontic brackets, in addition to long periods of treatment, have been shown to exert greater influence on the degradation process and friction force of these devices when compared to the actual oral pH.^17^ There are reports on the effects of prophylactic techniques in conventional brackets without^13^ and after clinical use,^18^ but little has been said about the effects of prophylactic techniques on debris buildup in self-ligating brackets after clinical use, compared with conventional brackets.

Our results suggest cleaning with air-powder polishing is an effective clinical method to control debris buildup in the oral environment with orthodontic brackets. Similar results have been found for cleaning conventional brackets^18^ and orthodontic arches.^12^ ^,^ ^16^

For both conventional and self-ligating brackets, a single application of air-powder polishing on slot surfaces promoted efficient removal of debris. According to recent studies, patient's motivation associated with periodic cleaning^19^ of bracket slot and surface arches can minimize the impact of the degradation process and increase friction in orthodontic treatment.

The brackets used as sample in this study were debonded from patients at the end of orthodontic treatment. This methodology aimed at investigating the results of real clinical conditions. It is known that since its adhesion, orthodontic bracket begins to be affected by the oral environment. Biological factors include plaque buildup, saliva, erosive drinks with carbonic acid and bacteria living in the oral cavity. There is evidence of the unquestionable influence of *Actinomyces viscosus* on the corrosion behavior of Ni-Cr alloys.^20^ Mechanical factors comprise tooth brushing, orthodontic activities (arch removal and placement at each monthly visit), and friction between bracket and arch.^21^

Due to those factors, conflicting results can be found in the literature concerning *in vitro* studies of bracket dynamics,^2^ ^,^ ^3^ ^,^ ^11^ ^,^ ^13^ ^,^ ^22^ in addition to clinical or *ex vivo* studies.^6^ ^,^ ^9^ ^,^ ^12^ ^,^ ^14^ Analyses of retrieved brackets highlight the need to reassess the properties and clinical behavior of brackets during treatment, so as to make appropriate treatment decisions.^21^

Changes on bracket surfaces can compromise the dimensional accuracy of the slots, which can affect the interplay between arch and bracket. Bracket performances, such as torque expression, angulation (tip) and rotation control, can be reduced as a result,^21^ in addition to having friction levels increased between bracket and orthodontic wire.^22,23^

The results of this study reveal that debris buildup, observed at the end of orthodontic treatment, was higher for self-ligating brackets compared to conventional ones, thus corroborating previous reports of greater debris buildup in self-ligating brackets after clinical use.^22^ ^,^ ^24^ ^,^ ^25^ The monthly replace of used ligatures with new ones, frequently over fixed orthodontic treatment with conventional brackets, may be related to the reduced overall level of debris accumulated on conventional brackets. Therefore, the greatest debris buildup can also be associated with the proper bracket clip lock mechanism, which, unlike elastomers in the conventional system, is not renewed.

In addition to increased friction,^16^ ^,^ ^13^ the presence of debris and plaque may contribute to enamel demineralization around brackets. Although our results showed greater debris buildup in self-ligating brackets, one study^14^ comparing bacterial composition around self-ligating and conventional brackets identified that self-ligating appliances promoted reduced retention of bacteria compared to conventional brackets with elastomeric ligature. However, a recent study has shown that there is no evidence of a possible influence of bracket design (conventional or self-ligating) on colony formation and bacterial adhesion.^24^

All patients were treated by the same orthodontist, which decreased the risk of bias. Prophylactic basic care was the same in patients with conventional brackets and those with self-ligating ones. Also, it can be inferred that the brackets examined probably have smaller debris buildup than in practices in which regular cleaning of orthodontic appliances is not carried out.

Other factors that can influence the levels of debris buildup are patient's oral care habits and the total time that brackets have remained in the oral environment, taking into consideration that both affect biofilm buildup in the oral cavity.^9^ ^,^ ^10^ ^,^ ^26^ ^,^ ^27^ Longitudinal studies should take into consideration these factors and take a deeper look at the relationship between the consequences of debris levels with mechanical and chemical processes involved in orthodontic treatment.

## CONCLUSION

At the end of orthodontic treatment, ceramic self-ligating brackets showed higher levels of debris compared to conventional metal brackets. Prophylaxis of orthodontic brackets with air-powder polishing was effective in debris reduction in both self-ligating and conventional brackets.
